# Smart Sensor Network Architecture with Machine Learning-Based Predictive Monitoring for High-Complexity Computed Tomography Systems

**DOI:** 10.3390/s26092619

**Published:** 2026-04-23

**Authors:** Arbnor Pajaziti, Blerta Statovci

**Affiliations:** Faculty of Mechanical Engineering, University of Prishtina, 10000 Prishtina, Kosovo; arbnor.pajaziti@uni-pr.edu

**Keywords:** smart sensor networks, predictive analytics, machine learning, computed tomography, fault diagnosis

## Abstract

This study addresses the need for intelligent condition monitoring in high-complexity medical imaging systems by proposing a smart sensing architecture for the Revolution EVO Computed Tomography (CT) scanner. Ensuring operational reliability and minimizing unexpected downtime remain critical challenges in advanced CT platforms, motivating the integration of distributed sensing and data-driven analytics. System logs spanning August 2024 to October 2025 were processed into 10-min intervals and converted into a structured dataset comprising 76 features derived from operational events, scanning parameters, and temporal dynamics. Two supervised learning models, the Support Vector Machine (SVM) and Artificial Neural Network (ANN), were trained to identify abnormal operating conditions. Both models delivered excellent classification performance, achieving an accuracy of 0.973. The SVM demonstrated balanced precision, recall, and F1-score metrics of 0.973, while the ANN outperformed in ranking and sensitivity to anomalies with an AUROC of 0.993 and an AUPRC of 0.976. This framework highlights the potential of sensor-driven machine learning in enabling early detection of system anomalies and optimizing maintenance planning within clinical CT environments.

## 1. Introduction

The reconstruction of images from projections has been investigated since the 1940s, long before the development of modern computational technology [1]. In 1940, Gabriel Frank filed a patent outlining the basic principles of what is now recognized as CT, describing methods for creating sinograms and applying optical back projection for image reconstruction [2]. Although early images were blurred, this work established the foundations for modern tomographic devices [2]. Two decades later, in 1961, neurologist William H. Oldendorf conducted a pioneering experiment to assess whether transmission measurements could identify internal structures in dense media, demonstrating early differences in material absorption using a rotating phantom and a NaI detector [3]. Subsequent advances include the introduction of transverse radioisotope tomography by Kuhl and Edwards in 1963 [4] and the formalization of the mathematical basis of reconstruction via the Radon transform in 1917 [5,6]. A landmark development occurred in 1967 when Hounsfield constructed the first CT scanner, leading to the first clinical installation in 1971 and the Nobel Prize for Hounsfield and Cormack in 1979 [2]. Modern CT imaging is based on acquiring X-ray projections from multiple angles and reconstructing the spatial distribution of tissue attenuation coefficients [7,8] by relying on fast detectors, precise X-ray sources, and advanced numerical algorithms [8].

CT is a fundamental medical imaging modality that enables the reconstruction of internal anatomical structures from X-ray projections acquired at multiple angles [7,8]. Modern CT systems rely on the precise coordination of X-ray sources, detectors, and mechanical components, combined with advanced reconstruction algorithms, to produce high-resolution images used for clinical diagnosis [8].

Over the past decades, significant progress has been made in image reconstruction techniques and hardware development, leading to improved image quality and reduced acquisition times [7,8]. However, the increasing complexity of CT systems introduces new challenges related to operational monitoring, system reliability, and maintenance management [9,10,11]. Failures in critical components such as the X-ray tube, detectors, or gantry rotation mechanisms can significantly degrade image quality and lead to costly downtime [10,11].

Traditional maintenance strategies in CT systems are typically based on fixed schedules or reactive interventions, which do not fully exploit the large amount of sensor and operational data generated during system usage [12,13]. In recent years, ML approaches have been increasingly applied to predictive maintenance and anomaly detection in complex systems, demonstrating promising results in identifying patterns associated with system degradation and failures [14,15]. In medical imaging contexts, the integration of intelligent sensor networks and data-driven models has enabled more efficient monitoring and improved system reliability [16].

Despite these advancements, several challenges remain insufficiently addressed. Existing approaches often rely on large, labelled datasets, lack interpretability, or are not easily adaptable to real-time monitoring environments. To further this study within the existing body of research, a more detailed analysis of recent contributions in predictive maintenance of medical imaging systems is provided. Wang et al. [9] proposed an anomaly detection framework based on Internet of Medical Things (IoMT) data, demonstrating the potential of ML for identifying operational irregularities in CT systems. However, their approach relies heavily on labelled datasets and does not address real-time monitoring constraints.

Zhou et al. [10] introduced a data-driven maintenance model for healthcare facilities, showing improvements in maintenance planning. Nevertheless, the study lacks integration of heterogeneous sensor data and does not consider system-level complexity. Similarly, Tang et al. [11] focused on the classification of X-ray tube malfunctions using artificial intelligence methods, but their approach is limited to specific components rather than the full CT system.

More recent work by Zhong et al. [12] explored AI-driven predictive maintenance in radiology environments, highlighting the effectiveness of Deep Learning (DL) models. However, challenges related to interpretability, data imbalance, and deployment in real-time clinical environments remain insufficiently addressed.

To provide a clearer comparison of existing approaches and highlight their limitations, a summary of related work is presented in Table 1.

As summarized in Table 1, existing approaches demonstrate significant progress in predictive maintenance of medical imaging systems. However, these methods are often limited by the lack of real-time capability, insufficient integration of heterogeneous sensor data, and reduced interpretability. Additionally, challenges such as data imbalance and system complexity remain insufficiently addressed. These observations highlight the need for a comprehensive and scalable solution, as proposed in this study.

These limitations indicate a clear research gap in the development of integrated, interpretable, and real-time capable predictive maintenance systems for complex CT platforms. The present study addresses this gap by proposing an SSN architecture combined with ML models for system-level monitoring and anomaly detection.

The approach combines statistical anomaly detection with Supervised Learning (SL) models to enable early identification of system irregularities and support proactive maintenance strategies.

The main contributions of this work can be summarized as follows:

- A data-driven framework for monitoring and anomaly detection in CT systems based on real operational data.
- The integration of statistical methods and ML models for improved detection performance.
- A comparative analysis of different predictive models for maintenance optimization.
- A scalable approach suitable for real-time system monitoring and deployment.

The remainder of this paper is organized as follows. Section 2 presents the theoretical background of the applied methods. Section 3 describes the materials and methods, including data collection and model development. Section 4 presents experimental results. Section 5 discusses the findings and limitations of this study. Finally, Section 6 concludes this paper and outlines directions for future work.

## 2. Preliminaries

This section presents the theoretical background underlying CT image reconstruction and the ML methods employed in this study. The aim is to provide the necessary mathematical and conceptual foundations for the proposed approach.

### 2.1. Image Reconstruction in CT

In CT, the objective is to reconstruct the spatial distribution of X-ray attenuation coefficients within the scanned object. These coefficients represent the extent to which tissues absorb X-ray radiation. The CT system acquires projections from multiple angles, where each projection corresponds to the integral of attenuation values along a given ray path [7,8].

Image reconstruction methods aim to solve this inverse problem using either analytical or iterative techniques.

### 2.2. Algebraic Reconstruction Technique (ART)

The Algebraic Reconstruction Technique (ART) is an iterative method that estimates the image by successively refining the solution of the linear system [7]. Each projection contributes to updating the estimated image values based on the discrepancy between measured and calculated projections.

The update rule for ART can be expressed as

(1) $$p_{i}=\sum_{j=1}^{N}a_{ij}\cdot x_{j}$$

where

p_i_ is the measured value of the ith projection.

a_ij_ represents the weight or length of the segment that ray i passes through cell j.

x_j_ is the unknown value that we want to determine.

The system of equations is written as

(2) P=A·x

This is solved iteratively with

(3) $${x_{j}}^{\left(k+1\right)}={x_{j}}^{\left(k\right)}+\lambda \cdot \left[\frac{p_{i}-\sum_{j}a_{ij}{x_{j}}^{\left(k\right)}}{\sum_{j}{a_{ij}}^{2}}\right]\cdot a_{ij}$$

where $x_{j}^{\left(k\right)}$ is the current estimate, $p_{i}$ is the measured projection, $a_{ij}$ represents the system matrix elements, and λ is a relaxation parameter controlling convergence [7].

ART is particularly useful in scenarios with limited or noisy data, although it is computationally more intensive compared to analytical methods.

### 2.3. Filtered Back Projection (FBP)

Filtered Back Projection (FBP) is one of the most widely used analytical reconstruction methods in clinical CT due to its computational efficiency and robustness under standard acquisition conditions [7,8].

The method consists of two main steps. First, the measured projections are transformed into the frequency domain and filtered to enhance high-frequency components:

(4) P(v,θ)

where v represents the frequency variable and ∣v∣ corresponds to the ramp filter [7].

The filtered projections are then transformed back to the spatial domain and distributed along their original acquisition paths using back projection:

(5) $$g\left(t,\theta \right)=F^{-1}[P(v,\theta )\cdot \left|v\right|]$$

**Figure 1:**
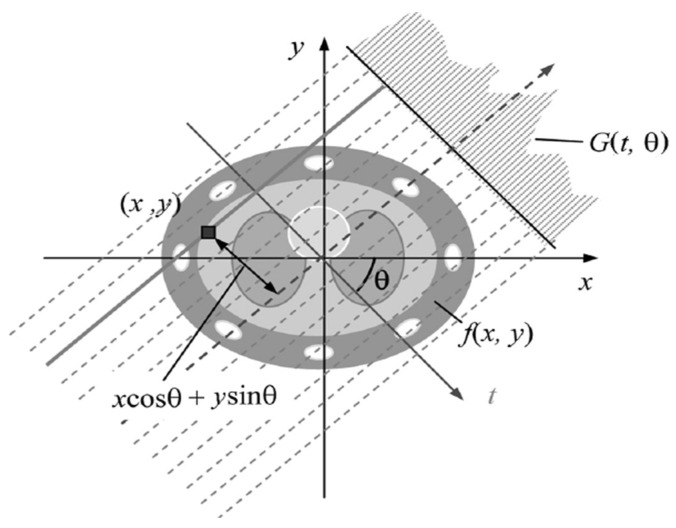
Illustration of the back projection process [7].

Although FBP is computationally efficient, it is sensitive to noise and motion artifacts [7,8]. In Figure 1, the geometric principle of image reconstruction through multiple projections at different angles is presented.

### 2.4. Fourier Slice Theorem

The Fourier Slice Theorem establishes a fundamental relationship between the Fourier transform of the projections and the Fourier transform of the reconstructed image. Specifically, it states that the one-dimensional Fourier transform of a projection at a given angle corresponds to a slice through the two-dimensional Fourier transform of the object at the same angle [8].

(6) $$F_{1}\left[P_{\theta }\left(\rho \right)\right]=F_{2}\left[f\left(x,y\right)\right]|\omega_{x}=\omega \cos\theta ,\;\omega_{y}=\omega \sin\theta \;$$

This principle provides the theoretical basis for analytical reconstruction techniques such as FBP and enables efficient implementation of reconstruction algorithms.

### 2.5. ML Models

In addition to classical reconstruction techniques, ML models are increasingly used for system monitoring and anomaly detection in complex systems [14,15]. In this study, two widely used SL approaches are considered: Support Vector Machines (SVMs) and Artificial Neural Networks (ANNs).

#### 2.5.1. SVM Models

SVMs are SL models used for classification tasks. The goal of the SVM is to determine an optimal decision boundary that maximizes the margin between different classes in the feature space. This property makes the SVM particularly effective in high-dimensional spaces and in cases where the number of samples is limited.

#### 2.5.2. ANN Models

ANNs consist of interconnected layers of processing units that can learn complex nonlinear relationships from data. By adjusting internal weights through training, ANN models can approximate highly nonlinear mappings between inputs and outputs, making them suitable for detecting subtle patterns in system behaviour.

The presented methods provide the theoretical foundation for both image reconstruction and data-driven analysis. While classical reconstruction techniques are essential for generating CT images, ML models enable advanced monitoring and predictive capabilities, which form the basis of the proposed approach in this study.

## 3. Materials and Methods

### 3.1. CT System Description

This study employs a combination of experimental and analytical methods to investigate the operation and maintenance of CT scanners, with a particular focus on the Revolution EVO model. The Revolution EVO scanner, manufactured by GE Healthcare (Chicago, IL, USA), represents a modern generation of volume CT systems designed to provide high-resolution imaging, reduced radiation dose, and improved clinical efficiency [17].

A detailed analysis of the primary CT scanner components, including the X-ray source, detectors, rotation mechanism, and data processing system, was conducted to identify key parameters influencing device performance and operational efficiency. The methodology was defined in sufficient detail to ensure reproducibility of the study [18,19,20,21,22,23].

### 3.2. Data Collection and Dataset

The dataset was collected from the system logs of the Revolution EVO CT scanner at the “Isa Grezda” Hospital in Gjakova, Kosovo, covering the period from August 2024 to October 2025. The data includes complete operational cycles, maintenance intervals, and variations in system workload.

The logs contain continuous records of system activity, including runtime events, scanning parameters such as DFOV, slice thickness, gantry tilt, and number of slices, as well as signals indicating scan initiation, termination, abort events, and data transfers. These logs provide a comprehensive representation of system behaviour and operational conditions.

### 3.3. Data Processing and Feature Extraction

The raw system log data were transformed into a structured dataset using fixed time windows of 10 min. Each time window represents a single sample capturing system activity within that interval.

The preprocessing pipeline included filtering irrelevant log entries and extracting relevant technical parameters from text-based logs. Key operational variables such as DFOV, slice thickness, gantry tilt, number of slices, and event-based signals were identified and processed.

Feature engineering was performed by aggregating the extracted data using statistical measures. These include mean values, event counts, and entropy-based indicators to capture variability and irregularities in system behaviour.

After preprocessing, a total of 272,948 events were extracted, resulting in a dataset of 13,468 samples with 76 features. This structured representation enables the application of ML models for anomaly detection.

**Figure 2:**
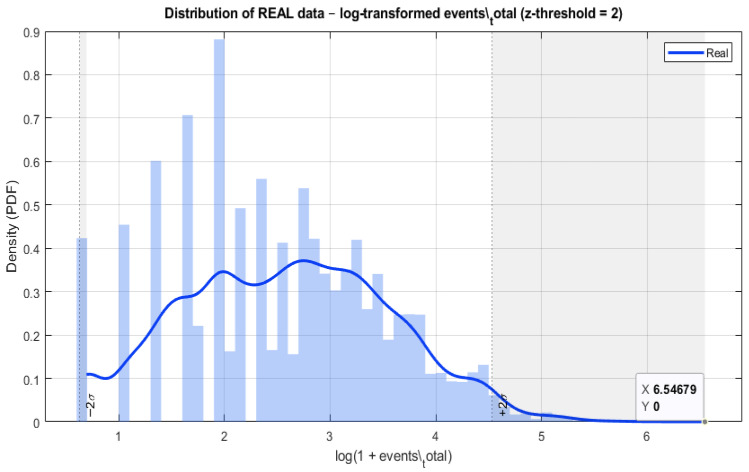
Distribution of the real data after logarithmic transformation for the variable “events_total”.

Figure 2 illustrates the distribution of the total number of events per time window after logarithmic transformation, highlighting the distinction between normal and abnormal operating regimes.

### 3.4. ML Methods

ML methods were applied to the processed dataset to develop predictive maintenance models. A neural network model was trained to identify potential defects and support maintenance optimization. The implementation was carried out in MATLAB R2021a, and all model configurations, training procedures, and parameter settings are documented to ensure full reproducibility of the computational analysis.

The dataset was divided into training and testing subsets using an 80–20% split to evaluate model performance. Standard evaluation metrics, including accuracy, precision, recall, F1-score, and the area under the Receiver Operating Characteristic curve (ROC-AUC), were used to assess the effectiveness of the implemented models. This evaluation ensures a reliable comparison of model performance and supports the validation of the proposed predictive maintenance approach.

#### 3.4.1. ANN Performance

An ANN was trained to model nonlinear relationships between system parameters and anomaly occurrence. The model consists of an input layer containing normalized features, two hidden layers with ReLU activation functions, and a sigmoid output layer representing anomaly probability.

The ANN achieved an ROC AUC of 0.993 and a PR AUC of 0.976, with an overall accuracy of approximately 97.3%. These results demonstrate the model’s capability to capture complex patterns associated with abnormal system behaviour.

**Figure 3:**
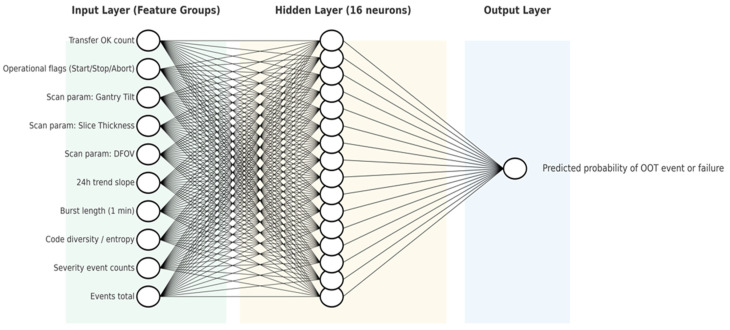
ANN Architecture for Predictive Maintenance of the Revolution EVO CT Scanner.

Figure 3 presents the architecture of the ANN model and the feature groups used as input.

#### 3.4.2. SVM Performance

A SVM model with a radial basis function kernel was developed as a complementary approach. The model was configured using balanced class weights to address class imbalance and optimized decision thresholds to improve classification performance.

The SVM achieved an ROC AUC of 0.996 and a PR AUC of 0.981, with an overall accuracy of 97.3%. Compared to the ANN, the SVM demonstrated higher precision, while the ANN achieved slightly higher recall. This indicates that the ANN is more sensitive to anomaly detection, whereas the SVM reduces false-positive predictions.

**Figure 4:**
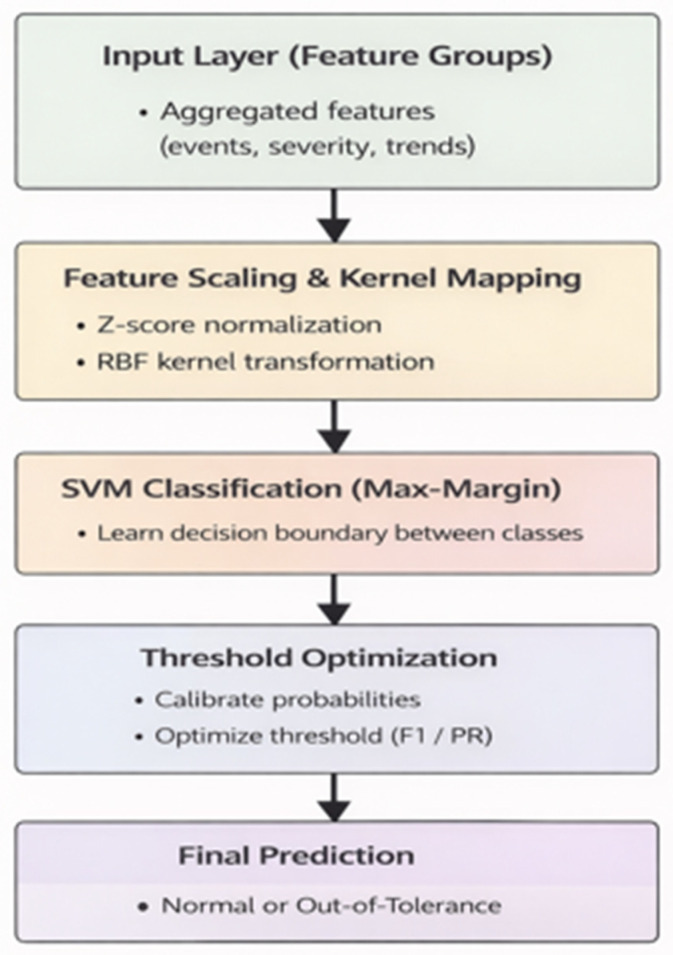
SVM Model Architecture for Predictive Maintenance of the Revolution EVO CT Scanner.

Figure 4 illustrates the workflow and architecture of the SVM model.

### 3.5. Experimental Setup

The proposed methodology enables systematic analysis of CT system behaviour and supports the identification of early indicators of component degradation. The results obtained from this approach are presented and evaluated in the following section.

To improve methodological clarity, Algorithm 1 summarizes the main steps of the proposed predictive maintenance pipeline [23].
**Algorithm 1.** Pseudocode of the proposed predictive maintenance pipeline for the Revolution EVO CT scannerInput: Raw CT system logs from 2024 to 2025 Output: Trained SVM and ANN models, evaluation results, and monitoring-ready outputs 1: Read all relevant CT log files 2: Remove duplicated log files 3: Parse log entries and extract event information:
      - timestamp
      - event code
      - severity
      - module and submodule
      - message content 
4: Filter data to retain only events from 2024 to 2025 5: Group events into fixed 10-min windows
6: Build a structured feature table for each window, including:
      - total event count
      - severity counts
      - module and code frequencies
      - entropy of event codes
      - burst activity
      - temporal trend features
      - scan and gantry parameters 
7: Save the generated feature table and feature catalogue 
8: Create surrogate anomaly labels using z-score-based rules on:
      - total events
      - severity ratio
      - burst activity
      - gantry tilt 
9: Select numerical features and preprocess the dataset:
      - replace missing values
      - standardize features

10: Split the dataset into training and testing subsets
        using a chronological 80 percent and 20 percent partition 
11: Train the SVM model with RBF kernel:
        - apply class weighting for imbalanced data
        - optimize classification threshold 
12: Train the ANN model:
        - use one hidden layer
        - apply threshold-based prediction 
13: Evaluate both models on the test set using:
        - accuracy
        - precision
        - recall
        - F1 score
        - ROC analysis
        - precision recall analysis 
14: Generate evaluation figures:
        - confusion matrix
        - ROC curve
        - precision recall curve 
15: Save trained models, reports, and output files 16: Return final models and performance results

**Figure 5:**
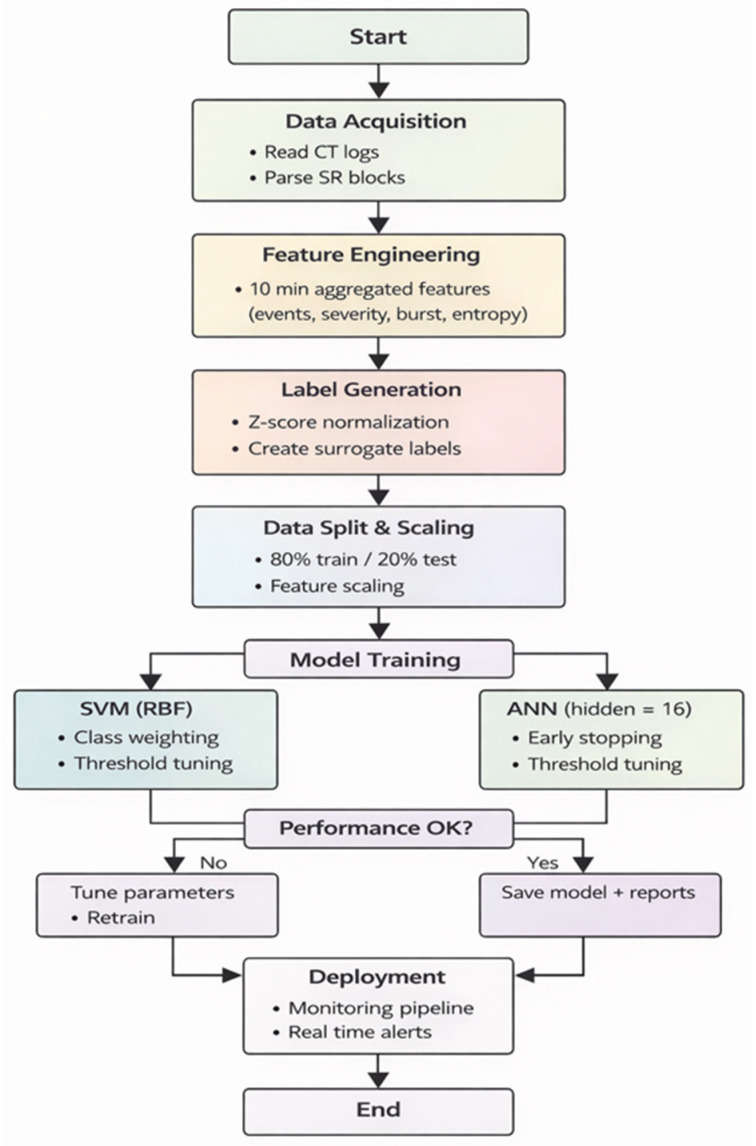
Flowchart of the pipeline of Predictive Maintenance in CT Revolution EVO (SVM + ANN).

The pipeline was implemented in MATLAB and supports periodic execution for continuous monitoring. Figure 5 presents the overall workflow of the proposed system, including data ingestion, feature generation, model training, and evaluation.

## 4. Results

This section presents the results of the proposed predictive maintenance framework applied to the Revolution EVO CT scanner. The approach integrates system log data, statistical analysis, and ML models to identify abnormal system behaviour and support proactive maintenance strategies.

### 4.1. Model Performance

The performance of the SVM and ANN models is summarized in Table 2. Both models achieved a high overall classification accuracy of approximately 97.3%.

The SVM model demonstrated balanced performance across all evaluation metrics, achieving precision, recall, and F1 score values of 0.973. In comparison, the ANN model achieved a precision of 0.91, a recall of 0.93, and an F1 score of 0.92.

Regarding ranking-based evaluation metrics, the ANN model achieved a higher Area Under the Receiver Operating Characteristic Curve (AUROC) of 0.993 and an Area Under the Precision Recall Curve (AUPRC) of 0.976. The SVM model achieved an AUROC of 0.973 and an AUPRC of 0.973. These results indicate that both models perform strongly, with slight differences in their classification behaviour.

### 4.2. Confusion Matrix Analysis

**Figure 6:**
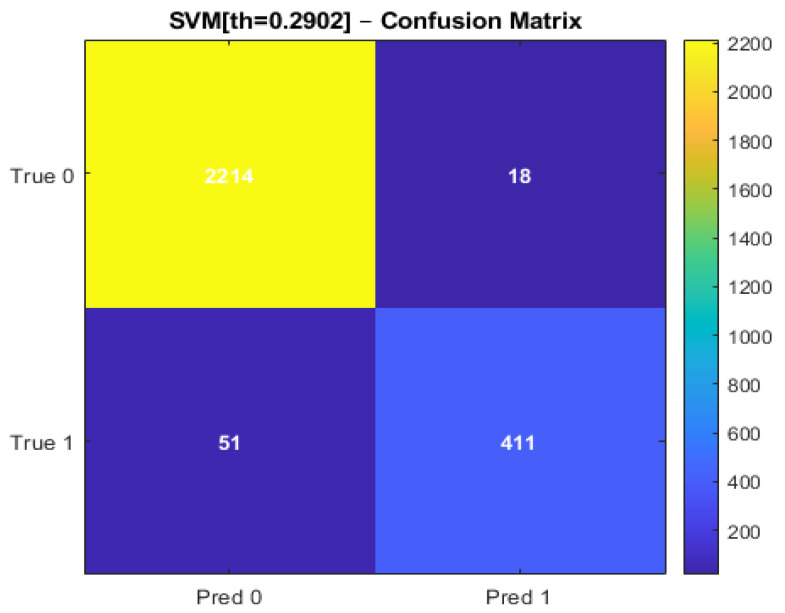
Confusion Matrix of the SVM.

**Figure 7:**
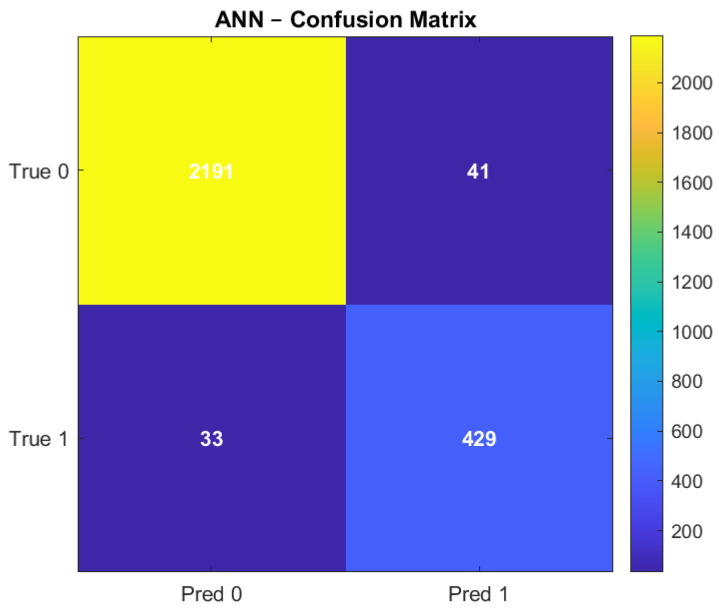
Confusion Matrix of the ANN.

The confusion matrices for both models are presented in Figure 6 and Figure 7.

The SVM model shows a lower number of false-positive classifications, indicating a more conservative classification behaviour. The ANN model, on the other hand, demonstrates a higher number of correctly identified anomalous instances, as reflected in a slightly higher number of true positives.

Both models maintain a low number of misclassifications overall, confirming their strong predictive capability.

### 4.3. Dataset Characteristics

The dataset statistics are summarized in Table 3. The data shows variations in system activity over time, with fluctuations in the number of events and anomaly occurrences across different months.

Periods with higher system workload are associated with increased event counts. Mid-2025 shows a noticeable increase in activity levels compared to earlier periods.

**Figure 8:**
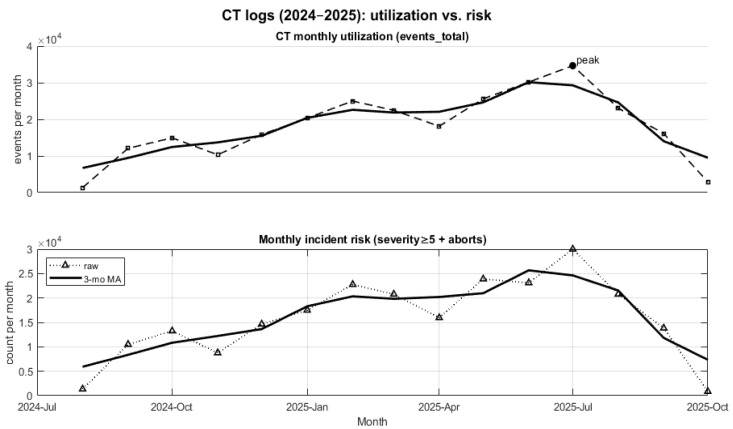
Relationship Between Monthly Scanner Workload and Incident Risk (2024–2025).

This relationship is further illustrated in Figure 8, which shows the temporal alignment between system workload and incident risk. The figure demonstrates that peaks in system usage coincide with higher anomaly rates, confirming that usage intensity is a key factor affecting system stability.

**Figure 9:**
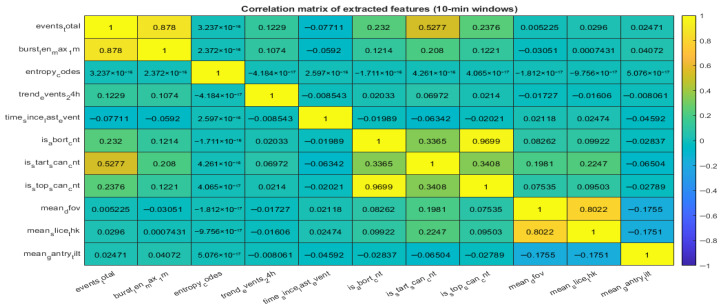
Correlation Matrix of Extracted Features (10-min Windows).

Additionally, the correlation matrix presented in Figure 9 highlights strong relationships between several key variables. For example, the high correlation between total events and burst activity indicates periods of intensive system load, while correlations between scan-related variables reflect operational dependencies within the system.

### 4.4. Feature Importance

**Figure 10:**
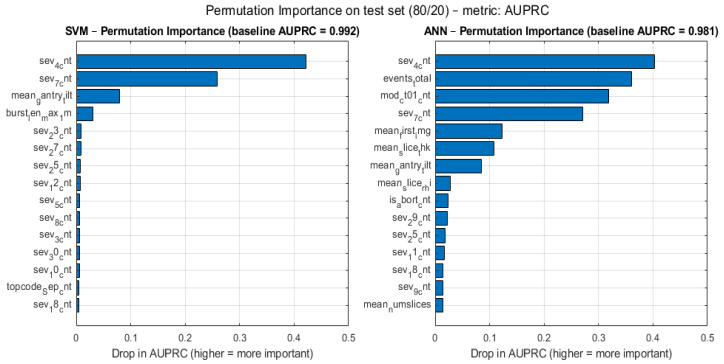
Permutation Importance for the SVM and ANN Models (test set, 80/20).

The contribution of individual features to model predictions is illustrated in Figure 10.

The results show that features related to high-severity events have the strongest influence on the classification outcome. Additionally, features capturing burst behaviour and frequency patterns also contribute significantly to the model performance.

Less influential features include lower severity event counts and static system characteristics.

### 4.5. Temporal Patterns of Anomalies

**Figure 11:**
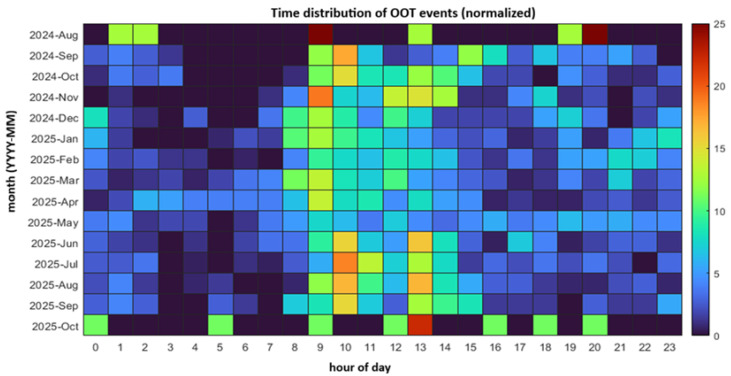
Temporal distribution of OOT (Out-of-Tolerance) events for the period 2024–2025.

Temporal trends in anomaly occurrences are presented in Figure 11.

The results indicate that anomalies are not uniformly distributed over time. Instead, they tend to occur more frequently during periods of increased system activity. Peak operational periods correspond to higher anomaly rates, suggesting a relationship between system load and anomaly occurrence.

### 4.6. Temporal Distribution of Prediction Errors

**Figure 12:**
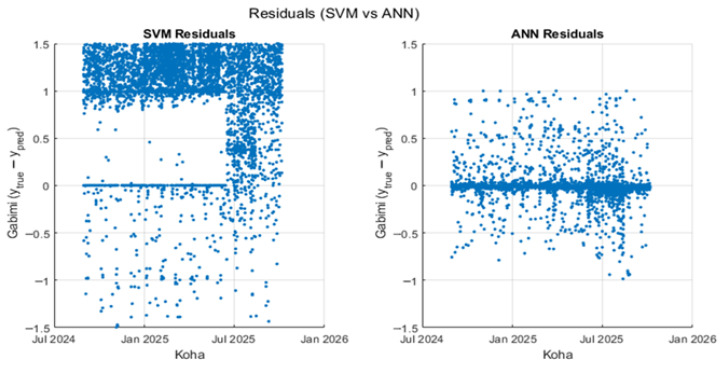
Temporal distribution of residuals for the SVM and ANN models.

The temporal evolution of prediction errors for both models is presented in Figure 12.

The results reveal differences in model behaviour across varying workload conditions. The SVM model exhibits larger deviations during peak-workload periods, indicating higher sensitivity to changes in system activity. In contrast, the ANN model maintains a more stable error distribution over time.

### 4.7. Threshold Sensitivity Analysis

**Figure 13:**
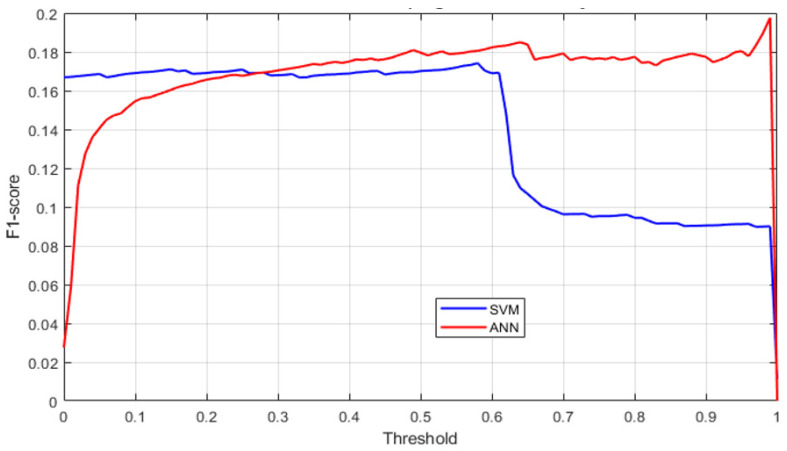
Sensitivity of the F1-score to the decision threshold for SVM and ANN.

The sensitivity of model performance to decision thresholds is illustrated in Figure 13, which shows the variation in F1 score for both models.

The results indicate that the ANN maintains stable performance across a wider range of thresholds, while the SVM is more sensitive to threshold selection. This highlights differences in model calibration and robustness.

### 4.8. Comparative Model Performance Visualization

**Figure 14:**
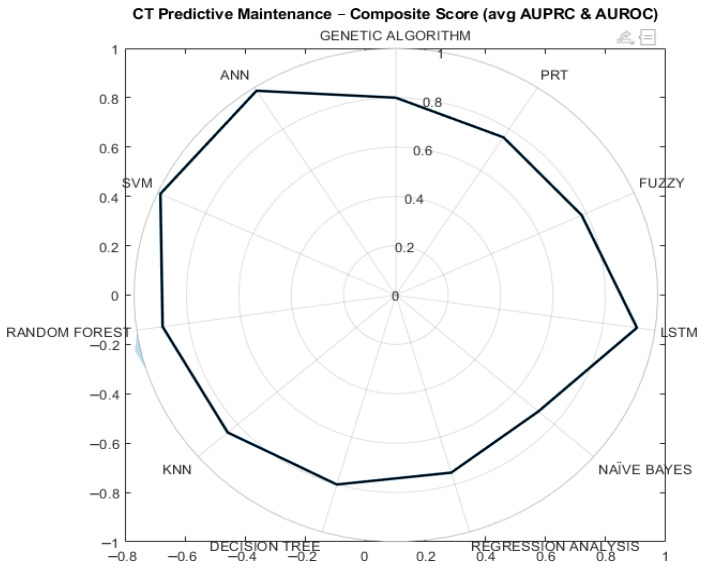
Relative comparison of ML model performance for PdM of the CT Revolution EVO (composite metric equal to the average of AUPRC and AUROC).

A comparative overview of model performance is presented in Figure 14, which shows a normalized radar chart based on a composite metric derived from AUPRC and AUROC.

The visualization confirms that both the SVM and ANN achieve strong performance across evaluation criteria, with slight differences in their behaviour. Both models outperform baseline approaches reported in the literature.

## 5. Discussion

This section interprets the results obtained from the proposed predictive maintenance framework applied to the Revolution EVO CT scanner, relating the observed model performance to the underlying system behaviour.

### 5.1. Comparative Model Performance

The results demonstrate that both the SVM and ANN models achieve strong overall classification performance, with similar accuracy levels. However, differences become evident when considering detailed evaluation metrics and model behaviour.

The SVM model exhibits balanced precision and recall, indicating stable and consistent classification performance. It follows a more conservative prediction strategy, reducing the number of false-positive classifications.

In contrast, the ANN model achieves higher recall and superior ranking performance, indicating improved capability in distinguishing between normal and anomalous instances across different decision thresholds.

### 5.2. Trade-Off Between Precision and Recall

The observed differences between the two models highlight a clear trade-off between precision and recall.

The SVM model prioritizes precision, making it suitable for environments where false positives may lead to unnecessary maintenance actions or increased operational costs.

On the other hand, the ANN model emphasizes recall, ensuring that a higher proportion of anomalies is detected. This characteristic is particularly important in predictive maintenance scenarios, where missed anomalies can result in system failures or increased downtime.

These findings suggest that model selection should be guided by the specific requirements and risk tolerance of the application.

### 5.3. Influence of Feature Engineering

The feature importance analysis indicates that high-severity event-related features play a dominant role in model predictions. This aligns with expectations, as critical system events are more likely to be associated with abnormal behaviour.

Additionally, features capturing burst patterns and temporal dynamics contribute significantly to predictive performance, highlighting the importance of incorporating time-dependent characteristics into anomaly detection models.

Less influential features include lower severity event counts and static system attributes, suggesting opportunities for feature optimization and dimensionality reduction.

### 5.4. Temporal Behaviour of Anomalies

The temporal analysis reveals that anomaly occurrences are closely related to system workload.

Periods of increased activity are associated with higher anomaly rates, indicating that system load plays a key role in the emergence of abnormal conditions. This suggests that predictive maintenance strategies should incorporate workload intensity as an important contextual factor.

### 5.5. Model Stability and Robustness

The analysis of prediction errors and threshold sensitivity highlights differences in model robustness.

The ANN model maintains a more stable error distribution over time, even during peak workload periods, indicating stronger robustness under dynamic operating conditions. In contrast, the SVM exhibits larger deviations during high-load intervals, suggesting greater sensitivity to changes in system activity.

Furthermore, the ANN demonstrates more consistent performance across varying decision thresholds, while the SVM is more dependent on careful threshold tuning. This indicates that the ANN may provide more reliable performance in real-world deployment scenarios.

### 5.6. Practical Implications

From a practical perspective, both models provide valuable capabilities for predictive maintenance.

The SVM model is better suited for environments where minimizing false positives is critical, such as systems with limited maintenance resources or high costs associated with unnecessary interventions.

The ANN model is more appropriate in safety-critical environments, where detecting as many anomalies as possible is essential to prevent system failures.

### 5.7. Limitations and Future Work

Despite the strong performance of both models, several limitations should be acknowledged.

The dataset may not capture all possible anomaly scenarios, which may limit the generalizability of the models. Additionally, the models were evaluated within a specific system context, and their performance may vary under different operational conditions.

Future work could focus on expanding the dataset, incorporating additional contextual and environmental features, and exploring hybrid or ensemble approaches that combine the strengths of both models.

## 6. Conclusions

This study presented a predictive maintenance framework for the Revolution EVO CT scanner based on ML and smart sensor data. The proposed approach integrates system logs and operational parameters to detect early deviations in system behaviour and support proactive maintenance strategies.

The experimental results demonstrate that both SVM and ANN models achieve high predictive performance, enabling reliable identification of anomalous operating conditions. These models can detect subtle changes in system behaviour that may precede mechanical or electronic failures, thereby allowing earlier intervention compared to traditional reactive maintenance approaches.

The findings indicate that predictive maintenance does not eliminate the occurrence of physical failures but significantly improves their management by reducing unexpected downtime and enhancing system reliability. Continuous monitoring enables the timely detection of critical conditions such as abnormal thermal behaviour, electrical instability, and ventilation inefficiencies, which are key indicators of potential system degradation.

From a practical perspective, the proposed framework contributes to improved operational efficiency, increased equipment availability, and enhanced safety in clinical environments. The results suggest that data-driven maintenance strategies can play a crucial role in optimizing the lifecycle and performance of advanced medical imaging systems.

Future work will focus on extending the proposed approach by incorporating more advanced models, including DL architectures for time series analysis, and by validating the framework across multiple devices and clinical environments. Additionally, integrating real-time deployment and explainable AI methods represents an important direction for improving trust and usability in medical applications.

This paper addressed the topic of predictive maintenance of the CT Revolution EVO scanner through ML and SSN. The main basis of the paper was the development of a proactive maintenance approach for the efficiency and long life of diagnostic CT, specifically the Revolution Evo.

In the implementation phase of the developed predictive maintenance approach, various ML models (SVM and ANN) were tested for the analysis of sensor data and the logs of the CT Revolution EVO system.

The results showed that the training models can identify small deviations in the system’s behaviour, which in real conditions would precede mechanical or electronic defects.

It was found that with the application of this method, the probability of unexpected breakdowns is significantly reduced, as the system can signal anomalies in real time, such as

- Unusual heating of the X-ray tube;
- Increased voltage in the DAS system;
- Decreased ventilation efficiency.

However, although physical breakdowns cannot be eliminated, they can be predicted and managed much more effectively through continuous data analysis.

Furthermore, it can be said that the implementation of predictive maintenance in CT Revolution EVO-type systems does not eliminate the possibility of defects, but significantly reduces their frequency, improving the safety, efficiency, and availability of the device in clinical settings.

For further developments, it is proposed to implement more advanced models, such as Autoencoder, Random Forest, or DL LSTM for time series-based prediction, and expand the application to other diagnostic devices.

## Figures and Tables

**Table 1:** Summary of related work on predictive maintenance in CT systems and related medical equipment.

Reference	Methodology	Application Area	Key Contributions	Limitations
Wang et al. [9]	ML + IoMT	CT anomaly detection	Integration of IoMT with ML for fault detection	Requires large, labeled datasets, limited real-time capability
Zhou et al. [10]	Data-driven predictive modelling	Healthcare equipment maintenance	Data-driven maintenance optimization	Limited integration of heterogeneous sensor data
Tang et al. [11]	AI-based classification	X-ray tube diagnostics	Detection of component-level failures	Focused only on a single component, lacks a system-level view
Zhong et al. [12]	DL	Radiology systems	Automated fault detection using deep models	Low interpretability, sensitive to data imbalance
Proposed Study	Smart sensing architecture with ML (SVM, ANN)	CT predictive maintenance	Real-time capable, interpretable multi-sensor analysis	Requires further validation across multiple systems

**Table 2:** Results for the SVM and ANN Models.

Model	Accuracy	Precision	Recall	F1-Score	AUPRC	AUROC
SVM (th = 0.29)	0.973	0.973	0.973	0.973	0.973	0.973
ANN	0.973	0.91	0.93	0.92	0.976	0.993

**Table 3:** Monthly Statistics of Events from the Revolution EVO CT Scanner Logs (November 2024–October 2025).

Month	Event Count	Severe Events	Aborts	Transfers OK	Scans (Started)	Unique Codes
August 2024	1349	0	1	96	118	218
September 2024	12,084	71	15	1138	1334	223
October 2024	14,936	468	32	1494	1758	225
November 2024	10,320	830	43	929	1092	225
December 2024	15,848	103	57	1122	1404	226
January 2025	20,350	914	90	1166	1373	223
February 2025	24,990	338	66	1150	1344	230
March 2025	22,452	249	37	1284	1435	229
April 2025	18,121	606	51	866	1011	212
May 2025	25,609	491	93	1280	1381	224
June 2025	30,167	1524	156	2024	2196	227
July 2025	34,652	1248	112	2538	2668	210
August 2025	23,039	696	167	1760	1938	229
September 2025	16,102	455	106	1277	1363	213
October 2025	2929	705	24	234	236	218

## Data Availability

https://github.com/blertastatovci/Smart-Sensor-Network-with-ML-Based-Predictive-Monitoring-for-CT-Systems (accessed on 5 March 2026).

## Abbreviations

The following abbreviations are used in this manuscript:

CT	Computed Tomography
FBP	Filtered Back Projection
ML	Machine Learning
SL	Supervised Learning
DL	Deep Learning
CMMS	Central Maintenance Management Systems
IOMT	Internet of Medical Things
ANN	Artificial Neural Network
OOT	Out of Tolerance
DFOV	Displayed Field of View
LSTM	Long Short-Term Memory
SVM	Support Vector Machine
DAS	Data Acquisition System
AUROC	Area Under the Receiver Operating Characteristic
AUPRC	Area Under the Precision–Recall Curve
SSN	Smart Sensor Network
